# Novel post-processing methods for infrared measurements on porous surfaces

**DOI:** 10.1038/s41598-025-33383-y

**Published:** 2025-12-23

**Authors:** Julian Härter, Grazia Lamanna, Rico Poser

**Affiliations:** Institute of Aerospace Thermodynamics, University of Stuttgart, Pfaffenwaldring 31, 70569 Stuttgart, Germany

**Keywords:** Infrared thermography, Emissivity, Self-pumping transpiration cooling, Energy science and technology, Engineering, Materials science, Physics

## Abstract

Infrared (IR) thermography is a key diagnostic tool for non-invasive measurements of surface temperatures. However, for porous materials, particularly those employed in self-pumping transpiration cooling systems, conventional IR techniques suffer from significant inaccuracies due to local emissivity variations caused by multiple materials, surface roughness, and presence of liquid in the porous media. This study presents and compares three post-processing methods to enhance the accuracy of IR temperature measurements on saturated porous surfaces. A constant emissivity approach, a blackbody radiator method, and a novel differential method are applied to an Isopropanol filled porous sintered bronze plate under controlled experimental conditions. The results reveal that the conventional constant emissivity method leads to the largest errors and broadest temperature distributions. The blackbody radiator method improves accuracy by introducing a reference emissivity point but retains significant uncertainties. The differential method, leveraging a distribution of reference temperatures, achieves the highest precision, effectively resolving local emissivity variations and minimizing deviations from thermocouple reference measurements. This method enables a detailed spatial characterization of surface temperatures, particularly capturing the thermal gradients associated with evaporation in self-pumping cooling regimes. Furthermore, the effect of liquid in metallic porous samples could be demonstrated and shows clear differences to ordinary metallic surfaces such as the border of the porous sample. These findings provide a refined framework and experimental validation for accurate thermal diagnostics of porous surfaces under two-phase flow conditions.

## Introduction

Infrared (IR) thermography has become an essential diagnostic tool for non-contact surface temperature measurements across a broad spectrum of technical applications ranging from aerospace engineering to material science. However, the accuracy of IR temperature measurements critically depends on the correct estimation of surface emissivities, which describes how efficiently a material emits thermal radiation relative to an ideal blackbody. For diffuse emitting surfaces with well-defined uniform properties, the emissivity can often be estimated with reasonable accuracy. However, for porous surfaces, this dependency becomes particularly problematic due to structural features such as surface roughness, internal cavities, pore size and distribution, pore shape and connectivity. These features significantly influence the porous medium’s physical properties, such as permeability, and may lead to inhomogeneous thermal properties^1–4^. Their intrinsic surface roughness and porosity lead to increased scattering, inter-reflection, and cavity radiation effects, all of which distort the effective radiative signature. Moreover, in self-pumping transpiration cooling, the presence of the liquid coolant also influences the local emissivity value.

Porous materials, especially those used in self-pumping transpiration cooling systems, are examples of the difficulty of determining surface temperatures using conventional IR thermography methods, as discussed in this work. In such systems, a liquid is passively drawn through a porous medium and evaporates at the hot surface, removing heat through phase-change. This introduces not only spatial temperature variations across the surface, but also an additional complication: the presence of liquid in between the porous cavities, which may partially obscure the surface or alter the radiative properties of the near-surface region. These effects can distort IR measurements if not properly accounted for, leading to significant underestimation or overestimation of local temperatures.

Moreover, partially varying surface conditions, such as the presence of wet and dry regions across the surface, lead to localized emissivity differences that are not accounted for by uniform calibration approaches. These spatial variations can introduce significant errors even under steady-state conditions, resulting in inaccurate surface temperature measurements. Another key challenge arises from the fact that porous structures made of metal often exhibit directional emissivity effects. Due to the complex geometry of internal pores and varying angles of emission, the measured radiance can be strongly dependent on the viewing angle, complicating the interpretation of IR data. Such anisotropic emission behavior has largely been overlooked in prior IR studies of porous surfaces.

Despite these known challenges, most existing studies on IR thermography applied to saturated porous materials continue to rely on the simplifying assumption of a constant emissivity across the surface^5–11^. Typically, a fixed emissivity value is applied, calibrated using thermocouple readings within the measurement region. For example, in Refs.^5–7^ and^11^ the authors investigated transpiration cooling with phase-change for sintered metallic porous plates. In Refs.^8^ and^10^, instead, the focus was on analysis of porous heat exchangers in scramjet combustor chambers. These works demonstrate the feasibility of IR thermography in such complex environments, but also highlight the limitations in capturing local temperature differences with high accuracy.

A comparable approach is observed in studies of porous materials with environmental relevance, such as soils, sands, and granular media. The authors in^12^ employed constant emissivity to monitor soil surface temperatures under varying moisture conditions. In the work of^13^ evaporation processes in sandy soils without accounting for local emissivity variations were investigated. Similarly, the authors of^14^ applied infrared thermography to study drying dynamics in porous media using uniform emissivity assumptions. These investigations primarily focused on the overall thermal behavior or moisture-related effects, with limited attention to spatial emissivity variations at the micro-scale. Additionally, while environmental studies commonly involve porous media like soil or sand, the structural and optical characteristics of these materials differ substantially from engineered porous metals. The scale of porosity, degree of surface roughness, and liquid content vary widely between environmental and technical applications, suggesting that emissivity assumptions from one domain may not be directly transferable to the other. Especially when analyzing non-diffuse metallic porous samples, as these have a strong directional dependence with regard to their emissivity^15^. This distinction has rarely been addressed explicitly in the literature.

The literature review revealed no studies employing methods to correct for local emissivity variations on saturated porous surfaces. This indicates a considerable untapped potential for enhancing temperature measurement accuracy, particularly in systems with phase-change-driven surface conditions like self-pumping transpiration cooling. In this study, three post-processing techniques are presented and compared to improve the accuracy of IR thermography on saturated porous surfaces. The first method assumes a constant emissivity, representing the simplest approach and corresponding to the method extensively applied in previous literature. The second utilizes a blackbody radiator placed in the measurement scene to calibrate the effective emissivity. The third and most advanced method is a differential method, which estimates local emissivity based on either spatial or temporal variations in surface temperature or radiance. This technique aims to resolve fine-scale emissivity differences and provide more accurate, spatially resolved temperature data. By applying these methods to saturated porous surfaces representative of transpiration cooling systems, we demonstrate how improved emissivity correction can reveal detailed temperature variations across the surface, enabling better insight into heat transfer processes, evaporation dynamics, and material behavior. In addition, we investigate the role of liquid in the porous sample and its influence on the infrared images. The outcomes of this work provide a valuable foundation for accurate thermal diagnostics in applications where porous media and liquid interactions play a critical role.

## Results

**Fig. 1:**
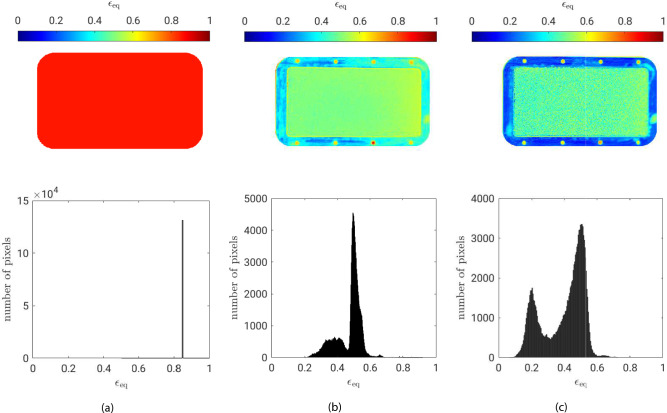
Variation and histogram of the equivalent emissivity $\epsilon _{eq}$ at $T_{\text {flow}}=353 \,\hbox {K}$: **(a)** constant emissivity method, **(b)** blackbody radiator method and **(c)** differential method. The upper figures represent a top view with the flow from left to right and a fully saturated porous sample under quasi-steady evaporation.

Three different post-processing techniques for improving the accuracy of IR thermography on saturated porous surfaces are compared in this study. The corresponding equivalent emissivity $\epsilon _{\text {eq}}$ distributions for the saturated porous surface and the surrounding sample holder are presented in Fig. 1. For the method applying a fixed emissivity across the entire measurement area (Fig. 1a), no local emissivity differences are observed, as expected. A global equivalent emissivity of $\epsilon _{eq} = 0.85$ was chosen to match the reference thermocouple temperatures during calibration. In contrast, the other two methods provide spatially resolved emissivity distributions by attempting to capture local variations across the surface. As shown in Fig. 1b and Fig. 1c, clear differences in emissivity are evident between the saturated porous sample and the sample holder. In both cases, the emissivity of the plane copper sample holder is significantly lower than that of the saturated porous material. However, the two methods also exhibit distinct patterns in emissivity distribution not only on the porous sample but also on the holder itself. With the differential method (Fig. 1c), the emissivity distribution across the sample holder is more pronounced compared to the blackbody radiator method (Fig. 1b). While the peak emissivity of the porous sample remains similar for both methods, differences emerge in the width of the distribution. The differential method demonstrates enhanced spatial resolution of local emissivity, particularly on the copper holder. This improvement enables the identification of small-scale surface roughness effects and localized emissivity changes induced by mechanical processing of the material. An additional difference is observed in the blackbody radiator cavity within the sample holder, where the differential method identifies a substantially lower emissivity value (Fig. 1c).

**Fig. 2:**
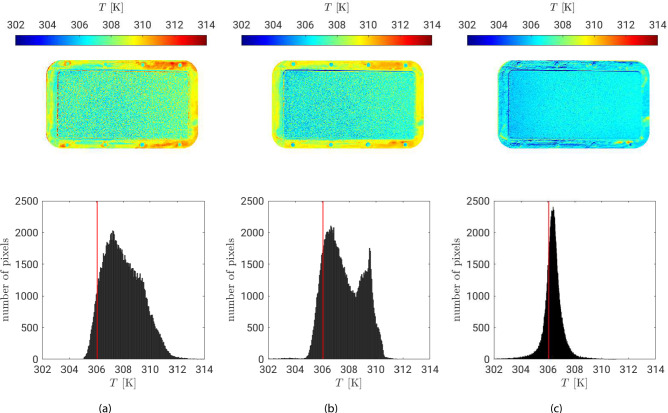
Variation and histogram of surface temperature $\epsilon _{eq}$ at $T_{\text {flow}}=353 \,\hbox {K}$: **(a)** constant emissivity method, **(b)** blackbody radiator method and **(c)** differential method. The upper figures represent a top view with the flow from left to right and a fully saturated porous sample under quasi-steady evaporation.

Evaluating the methods exclusively based on emissivity distributions is challenging in the absence of spectral surface analysis. Therefore, the focus shifts to the corresponding surface temperature distributions, calculated from the respective equivalent emissivities. Fig. 2 shows the measured temperatures from IR thermography based on the previously determined emissivities for the three different methods. In the histograms for the different methods, the average reference temperature of the thermocouples inside the copper frame is also indicated by a red line.

The constant emissivity method (Fig. 2a) and the blackbody radiator method (Fig. 2b) produce wide temperature distributions over both the saturated porous sample and the sample holder. In the case of the blackbody radiator method in Fig. 2b, there is a separation of the temperatures from the porous sample to the housing by a second peak in the distribution. For the constant emissivity method and the blackbody radiator method, higher temperatures are measured on the copper housing. In contrast, the differential method (Fig. 2c) yields a narrow temperature distribution across the measurement area, with only a minor offset from the thermocouple reference. For all three methods, measurements using IR thermography lead to a distribution of higher temperatures than those measured by the thermocouples.

**Fig. 3:**
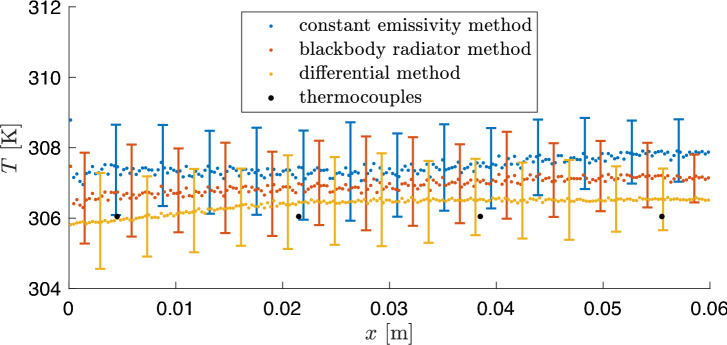
Comparison of the line-averaged surfaces temperatures of the porous medium with their axial distribution and the averaged thermocouple temperature for the different methods.

The differential method shows a slight change in temperature in the flow direction. Here, lower temperatures can be seen at the front of the saturated porous plate. This allows the localized cooling effect along the porous plate to be directly observed as a result of evaporation. For a more quantitative comparison, Fig. 3 shows the averaged temperatures distribution of the different methods in the axial flow direction only for the saturated porous surface without the copper housing. The bars show the distribution of temperatures depending on the respective *x*-position. For better visualization, not all bars are shown for every point and every method. In addition, the time-average temperature of the thermocouples in *x*-direction are also marked in black. All methods show a slight increase in temperatures in the direction of flow, whereby this is mostly visible for the differential method, as already mentioned. It is also noticed that the distribution of temperatures decreases slightly in the flow direction for all three methods.

As shown in the histograms in Fig. 2, a significantly larger standard deviation for the temperatures of the method with constant emissivity and the method using blackbody radiators is obtained. The smallest distribution for the temperature determination and also deviation from the thermocouples results for the differential method. As previously noted, the lowest temperatures are observed at the front of the porous plate, where the unsaturated hot air induces the highest local evaporation flux. However, a quasi-steady equilibrium between heat input and evaporative flux is quickly established, causing the surface temperature to stabilize after the initial drop.

This behavior is characteristic of self-pumping transpiration cooling systems with phase-change, as also reported in Ref.^6^. Their study showed that temperature distributions on the saturated porous surface were more uniform and stable compared to conventional transpiration cooling without phase-change. The present experiments further confirm these findings and demonstrate the effectiveness of the developed measurement techniques in reliably capturing and characterizing the performance of such two-phase cooling systems.

### Uncertainty analysis

Meaningful data analysis requires both proper data acquisition and a clear assessment of measurement accuracy. To this end, the measurement uncertainties associated with each method used are outlined below.

**Fig. 4:**
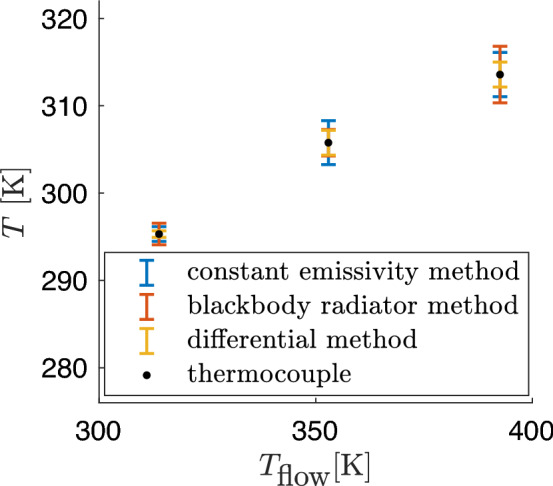
Comparison of the cumulative errors of the calculated average temperature of the thermocouple tips for the different methods.

All measurements inherently involve a certain degree of uncertainty. Fig. 4 shows the measurement accuracy for the different methods for certain flow temperatures $T_{\text {flow}}$. The temperatures determined using the different methods were compared with the mean value of the thermocouples (black). Only the local temperatures measured at the thermocouple tips were used in the infrared images. The thermocouples used were calibrated prior to installation, providing a measurement accuracy within ±0.2 K. For surface temperatures determined via IR thermography, both statistical and systematic uncertainties are taken into account and then combined into a cumulative error, as summarized in Fig. 1.

**Table 1:** Error (Systematic (Sys), statistical (Sta) and cumulative relative error (Cum)) of the calculated averaged thermocouple temperatures for the different methods.

		Flow temperature $T_{\text {flow}}$ [K]		
Methods	Error	313.8	352.9	392.5
Constant emissivity	Sys	0.798	2.229	- 0.144
	Sta	0.149	1.149	2.529
	Cum	0.283 %	0.852 %	0.860 %
Blackbody radiator	Sys	1.221	0.777	- 0.267
	Sta	0.108	1.279	3.228
	Cum	0.421 %	0.511 %	1.099 %
Differential	Sys	0.296	-0.999	0.199
	Sta	0.113	0.998	0.886
	Cum	0.127 %	0.483 %	0.315 %

## Discussion

The comparative analysis of the three post-processing methods reveals important insights into the challenges and potential improvements of IR thermography applied to saturated porous surfaces. The constant emissivity method, while straightforward and commonly used in literature, shows the largest deviation from the reference thermocouple readings, with wide temperature distributions. The measured surface temperatures of the copper holder for this method clearly differ from the reference thermocouples.

The blackbody radiator method offers an improvement by introducing a reference point with a well-defined emissivity, enabling partial compensation for unknown emissivity variations in the measurement field. Compared with the constant emissivity approach, this method allows a certain differentiation of the emissivity between the porous sample and the surrounding copper holder. Local differences in the wetting conditions on the pore scale and in the geometric features still lead to inconsistencies that cannot be fully resolved with a single blackbody reference. The observed deviations and temperature offsets indicate that this approach can partially correct the spatial emissivity variations within the porous region. However, the method shows its clear weakness in determining the correct emissivities for flat metal surfaces such as the copper surrounding.

The differential method demonstrates the highest accuracy and robustness, showing the smallest deviations and minimal temperature offsets relative to the reference measurements of the thermocouples. By employing two reference temperatures, this approach enables a direct estimation of local emissivity and effectively compensates for spatial variations as well as environmental and optical interferences. The observed fine-scale temperature gradients along the porous plate, particularly the lower temperatures at the front region due to evaporation, align well with the expected physical behavior of self-pumping transpiration cooling systems. Compared with the other methods, an almost homogeneous temperature distribution is shown for both the porous sample filled with liquid and its copper enclosure. This shows that this method can also be used to determine equivalent emissivities for emitters such as metals that are strongly dependent on the emission angle.

For the copper housing of the sample in particular, the constant emissivity method and the blackbody method show their weakness for such non-diffuse metal surfaces. This weakness appears to be less pronounced with the porous sample filled with liquid, as the deviation is much smaller here. Even if these samples are made of metal, the liquid in the cavities counteracts the angular dependence of the radiation towards a diffuse radiator. Indeed, the presence of liquid and vapor layer at the interface along the porous surface influences the IR measurement, since the emissivities of the liquid zones may differ significantly from the metal surrounding. This result clearly highlights the limitations of using a global equivalent emissivity or the blackbody radiator method on such complex coupled system, where local variations in surface conditions significantly influence radiative properties.

For the blackbody assumption, the comparison with the differential method shows an overestimation of the emissivity value for the blackbody cavity. This can be explained by assuming the non-consideration of the transmittance of the optical path $\tau _{\text {eq}} = 1$ for this method. The radiation emitted by the blackbody cavity that is absorbed by the camera is in reality much lower than the radiation emitted by the cavity. Compared with the emissivity distribution of the differential method, this leads to an overestimation of the determined emissivity.

Across all methods, a slight temperature discrepancy between the IR measurements and the thermocouples was consistently observed. This offset is most likely attributable to a small stem effect in the thermocouples, which causes a heat conduction along the thermocouples. Because the thermocouple protrudes toward the ambient environment, a small amount of heat is continuously conducted away from the junction along the thermocouple stem. Even a very small heat loss on the order of a fraction of a mW is sufficient to slightly cool the junction due to the long geometry of the thermocouple. With this, a small temperature difference of about ${0.5}\,\hbox {K}\,\hbox {to}\,{1}\,\hbox {K}$ between the thermocouple reading and the IR measurement is consistent with the expected stem effect of the thermocouple. As a result, IR thermography captures a higher surface temperature, while the thermocouple measurements show a lower temperature due to this gradient. A possible compensation could be achieved by isolating the thermocouples that extend below the copper holder.

Comparing the estimated errors of the individual methods, it can be seen that the differential method has the lowest relative error for different boundary conditions. But also the other two methods do not show large relative errors. It must be clearly stated that these errors only relate to the measuring range of the thermocouple tips. The averaging of the seven thermocouples used is probably the reason for the large systematic differences of the different methods. For a more precise error analysis of the various methods, the entire surface of the porous sample should also be taken into consideration in future. Due to the complex surface and, in particular, due to the presence of the liquid, there are considerably greater discrepancies with these methods. Consequently, the assumption of a constant emissivity and the blackbody reference would necessarily lead to large errors and thus to an inaccurate evaluation of the surface temperature distribution. This demonstrates that the differential method not only improves absolute temperature accuracy but also offers superior spatial resolution, capturing the critical thermal gradients necessary for a detailed understanding of local heat and mass transfer processes.

Overall, these findings emphasize that accurate temperature characterization of saturated porous surfaces, particularly under two-phase conditions, requires advanced emissivity correction strategies. Among the methods studied, the differential approach proves to be the most reliable in capturing local temperature variations with reduced uncertainty. The improved measurement precision and spatial detail achieved with this method can significantly enhance experimental investigations of complex porous media.

## Methods

### Test facility

**Fig. 5:**
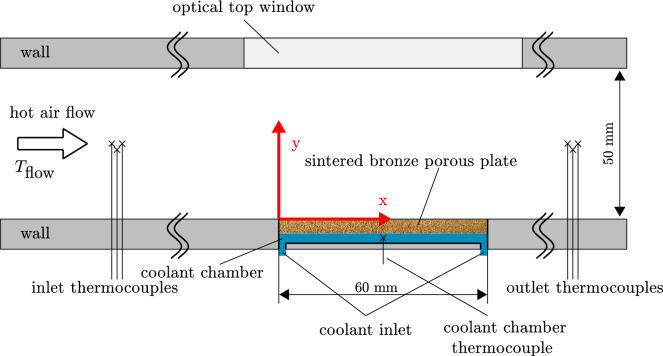
Schematic drawing of the measurement section.

The self-pumping transpiration cooling system was tested in a steady-state wind tunnel using high-temperature air. The wind tunnel setup was identical to that used in Ref.^16^, as shown in Fig. 5. Dry air was compressed by an *Atlas Copco type GA22*. The compressed air was heated by an electrical heater from *Leister type 10000*, to precisely control the temperature in the channel. Additionally, the flow conditions are regulated by a hydraulic valve and monitored using a mass flow meter. The cross section of the test section was rectangular, with an area of $200\,\hbox {mm}\, \times \, {50}\,\hbox {mm}$. A sintered bronze porous plate was installed at the bottom of the channel, with its top surface aligned parallel to the inner wall of the wind tunnel. The selection of the porous material follows the criteria outlined by^17^. The porous plate features an average pore diameter of ${55}\,\upmu \hbox {m}$ , with dimensions of ${60}\,\hbox {mm}\,\times \,{30}\,\hbox {mm}$ and a thickness of ${5}\,\hbox {mm}$. Further information on the porous material used can be found in Fig. 2. Isopropanol was used as the cooling liquid in this work. To enable infrared thermography, a zinc selenide window was used as the optical top cover, chosen for its high transparency in the infrared wavelength range of the used infrared camera.

**Table 2:** Parameters of the used porous medium.

Porous probe	Porosity $[\%]$	Permeability $[\hbox {m}^{2}] \times 10^{-12}$	Pore diameter $[\upmu \hbox {m}]$
B80	$39 -43$	80	55

**Fig. 6:**
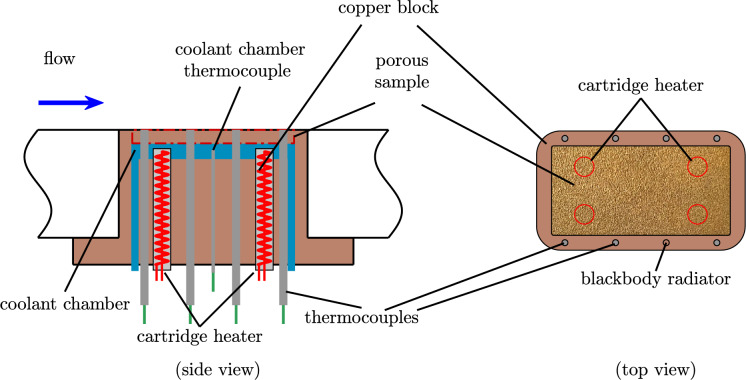
Side and top view of the copper sample holder block for the in-situ calibration for infrared measurements.

For this test campaign, a Reynolds number of $\text {Re} = {4500}$ was chosen as a reference case for turbulent flow. To accurately capture the temperature boundary conditions, three thermocouples were placed at both the inlet and outlet of the square channel. The upstream thermocouples were positioned approximately ${870}\,\hbox {mm}$ upstream of the porous sample to avoid disturbing the downstream flow. Downstream thermocouples were located about ${280}\,\hbox {mm}$ behind the sample. All thermocouples are installed through the bottom wall and extend to the middle of the channel height. The first ${5}\,\hbox {mm}$ of each thermocouple is bent parallel to the wall in upstream direction. Additionally, one thermocouple was installed to measure the coolant liquid temperature, as shown in Figs. 5 and 6.

In this work, a special copper sample holder was used in order to be able to apply the different evaluation methods of IR thermography. This enables in-situ calibration of the IR thermography measuring region, which is necessary for all three methods. As shown in Fig. 6, the copper sample holder was used to secure the porous plate, selected for its high thermal conductivity. The holder is heated by four electric heating cartridges, each rated at ${100}\,\hbox {W}$, with the heating power regulated by a *Juchheim LTR 3500* controller to maintain the target temperature. Seven calibrated K-type thermocouples with their insulated measuring tips are embedded in the copper block and decoupled from the flow at the top by means of heat-conducting paste. One of the thermocouple holes remains empty and serves as a blackbody radiator reference in the measurements. Thanks to the high thermal conductivity of copper, it is reasonable to assume thermal equilibrium is reached throughout the system. If the temperature is kept constant with the heating cartridges over an extended period, uniform temperatures are expected in the entire block, the porous sample, and the cooling liquid.

### Infrared thermography measurements

The surface temperature distribution of the porous medium and its surrounding holder were captured using an *InfraTec ImageIR 8340 hp MS* infrared camera, with an *lnSb* detector for the spectral range from 1.5 to ${5.7}\,\upmu \hbox {m}$ and a resolution of 640 x 512 pixels. All post-processing approaches are grounded by modeling the radiative intensity $I_{\text {cam}}$ recorded by the infrared camera, expressed as the sum of two main contributions:

1 $$\begin{aligned} I_{\text {cam}} = \sigma T_{\text {cam}}^4= \tau _{\text {eq}} (\epsilon _{\text {eq}} \sigma T^4) + I_{\text {noise}} \, , \end{aligned}$$

where $\sigma$ is the Stefan-Boltzmann constant. In this expression, $\tau _{\text {eq}}$ accounts for all transmission losses through the gaseous medium and optical components of the setup. The term $I_{\text {noise}}$ includes all unwanted contributions from the surroundings, such as thermal radiation from optical elements (lenses, filters, windows) and scattered radiation.

The parameter $\epsilon _{\text {eq}}$ represents the equivalent emissivity of an real emitter whose radiation matches that of the bronze/liquid surfaces and its copper holder. For the measurement setup described in this paper, this term will be examined in more detail below. The starting point is the equation for determining the spectral directional emissivity $\epsilon _{\lambda }$ of a real body as follows:

2 $$\begin{aligned} \epsilon _{\lambda }(T, \lambda , \Theta , \varphi ) = \frac{L_{\lambda }(T, \lambda , \theta , \varphi )}{L_{\lambda ,b}(T, \lambda )} \, , \end{aligned}$$

where $L_{\lambda }$ is the spectral radiative intensity emitted by the porous surface and the copper holder and $L_{\lambda ,b}$ is the corresponding spectral radiative intensity emitted by a blackbody. Based on the definition, $\theta$ is the polar angle, and $\varphi$ the azimuth angle. Integrating these spectral radiative intensities over there relevant solid angle leads to a fraction $\beta$ of absorbed to emitted spectral emissive power $M_{\lambda }$.

3 $$\begin{aligned} \beta = \frac{\int _{0^\circ }^{15^\circ } \epsilon (T, \lambda , \theta , \varphi ) L_{\lambda }(T, \lambda , \theta , \varphi ) \cos {\theta } \,\textrm{d}\Omega }{\int _{0}^{2\pi } L_{\lambda ,b}(T, \lambda ) \cos {\theta } \,\textrm{d}\Omega } = \frac{\int _{0^\circ }^{15^\circ } \epsilon (T, \lambda , \theta , \varphi ) L_{\lambda }(T, \lambda , \theta , \varphi ) \cos {\theta } \,\textrm{d}\Omega }{M_{\lambda ,b}(T, \lambda )} \end{aligned}$$

Here, $\Omega$ denotes the solid angle subtended by the distance of the infrared camera to the measuring region and its lens. The black body as a diffuse radiator is independent of the solid angle and thus becomes a hemispherical quantity. By further integrating over the spectral range to which the infrared camera is sensitive, the equivalent emissivity is obtained as:

4 $$\begin{aligned} \epsilon _{\text {eq}} = \frac{\int _{1.5 {\upmu {\textrm{m}}}}^{5.7 {\upmu {\textrm{m}}}} \int _{0^\circ }^{15^\circ } \epsilon (T, \lambda , \Theta , \varphi ) L_{\lambda }(T, \lambda , \Theta , \varphi ) \cos {\theta } \,\textrm{d}\Omega \,d\lambda }{\int _{0}^{\infty } M_{\lambda ,b}(T, \lambda ) \, \textrm{d}\lambda } = \frac{\int _{1.5 {\upmu {\textrm{m}}}}^{5.7 {\upmu {\textrm{m}}}} \int _{0^\circ }^{15^\circ } \epsilon (T, \lambda , \Theta , \varphi ) L_{\lambda }(T, \lambda , \Theta , \varphi ) \cos {\theta } \,\textrm{d}\Omega \,\textrm{d}\lambda }{\sigma T^4} \, . \end{aligned}$$

This definition reflects the ratio of emissive power emitted by the real saturated porous surface and its holder to that of a corresponding ideal blackbody. The total energy emitted by the blackbody can be simplified using Stefan-Boltzmann law.

The formulation Eq. 1 highlights that the apparent temperature measured by the infrared camera $T_{\text {cam}}$ does not directly represent the true surface temperature *T*. Accurate temperature estimation thus requires an appropriate correction for $\epsilon _{\text {eq}}$. The following sections describe the three different post-processing methods used to determine the equivalent emissivity $\epsilon _{\text {eq}}$ and from that the local surface temperatures.

#### Constant emissivity method

With this general approach, as has been used^5–11^, the infrared system was calibrated on the averaged temperature of the seven thermocouples of the sample holder. The internal emissivity of the camera was set to an equivalent emissivity $\epsilon _{\text {eq}}$ so that the measured surface temperatures of the thermocouple tips match the measured temperature of the thermocouples. This means that $\epsilon _{\text {eq}}$ is calibrated in Eq. 1 in such way that the temperature determined by the infrared camera corresponds to $T_{\text {cam}} =~T$. The calibration was performed with a heated copper block at a constant temperature of $T = 313.15\, \hbox {K}$ under isothermal steady conditions. This defined equivalent emissivity is constant for the entire measuring range of the camera and therefore does not take into account any material changes between the porous sample and the holder or the presence of liquid in the porous sample itself. Thus, there is no local resolution of the emissivity for this method and the surface temperatures determined by this method correspond to the values measured directly by the infrared camera.

#### Blackbody radiator method

In this method, we make use of the heatable copper holder of the porous sample. To determine the equivalent emissivity $\epsilon _{\text {eq}}$, we want to use a reference emitter with known properties. For the first validation of the measurements, the reference emitter has an emissivity close to 1, which was realized using a blackbody radiator in the sample holder. Since the transmission coefficient of copper $\tau _{co} = 0$, the reflectance $\rho _{co}$ can be calculated using the relation $\rho _{co} = 1 - \epsilon _{co}$. It was discovered that a simple cylindrical cavity with a large length *L* to radius *R* ratio can act as a blackbody radiator^18^. The advantage of this technique is that it allows direct comparison between the emissivity of the cavity and the surround surface. For this purpose, one empty thermocouple cavity with a radius of $R = 1.5 \, \hbox {mm}$ and a length of $L = 30 \, \hbox {mm}$ in the copper sample holder is used.

To determine the emissivity of the cavity, it is necessary to first estimate the emissivity of the copper surface. Since this value varies depending on its surface condition, e.g. oxidation, and the observation angle, a conservative value of $\epsilon _{co} = 0.1$ is applied in this study. From^18^ we obtain the reflectance $\rho _{c}$ for a cylindrical cavity with a plane bottom perpendicular to the axis:

5 $$\begin{aligned} \rho _{c} = \frac{\rho _{co}}{1 - \rho _{co}} \frac{1}{1 + (L/R)^2} \, . \end{aligned}$$

For the cavity dimensions of the copper sample holder, this results in an emissivity of the cavity of $\epsilon _{c} = 1 - \rho _{c} = 0.9997$. For a heated copper block with a temperature $T_{\text {ref}}$, we can assume that the entire copper block with the cavity and the saturated porous sample are in thermal equilibrium. Thus, it is possible to determine the equivalent emissivity at any point on the surface as a quotient of the measured radiation to the cavity.

6 $$\begin{aligned} \epsilon _{\text {eq}} = \frac{I_{\text {cam, ref}}}{I_{\text {cam, ref},c}} = \frac{T_{\text {cam, ref}}^4}{T_{\text {cam, ref},c}^4} \end{aligned}$$

Once the equivalent emissivity for a known reference temperature is determined, it can be used to evaluate the temperature distribution during transpiration cooling experiments. In these experiments, the porous medium is no longer actively heated. Instead, the measured surface temperature $T_{\text {exp}}$ reflects the balance between the heat transfer from free flow and the cooling effect caused by evaporation. By comparing the temperature measurements from the actual flow experiments with those from the reference case, the resulting temperature distribution can be analyzed.

7 $$\begin{aligned} \sigma T_{\text {cam, ref}}^4&= \tau _{\text {eq}} (\epsilon _{\text {eq}} \sigma T_{\text {ref}}^4) + I_{\text {noise}} \, , \end{aligned}$$

8 $$\begin{aligned} \sigma T_{\text {cam, exp}}^4&= \tau _{\text {eq}} (\epsilon _{\text {eq}} \sigma T_{\text {exp}}^4) + I_{\text {noise}} \, . \end{aligned}$$

By subtracting the above relation and solving for $T_{\text {exp}}$, it follows

9 $$\begin{aligned} T_{\text {exp}} = \root 4 \of {T_{\text {ref}}^4 -\frac{T_{\text {cam, ref}}^4-T_{\text {cam, exp}}^4}{\tau _{\text {eq}} \epsilon _{\text {eq}}}} \, . \end{aligned}$$

This approach assumes that both $\epsilon _{\text {eq}}$ and $I_{\text {noise}}$ remain constant between the reference case and the real experimental case, which is reasonable for small temperature differences. In addition, to determine the equivalent emissivity $\epsilon _{\text {eq}}$, it is assumed that the equivalent transmission $\tau _{\text {eq}}$ remains the same for these small temperature differences. To determine the temperature $T_{\text {exp}}$, we set $\tau _{\text {eq}} = 1$, as the transmission losses are already taken into account in the equivalent emissivity. It is important to note that these assumptions are valid under quasi-steady evaporation conditions at the surface. The surface emissivity is primarily determined by the properties of the porous material and the liquid retained within its pores. While vapor above the surface can slightly affect the transmissivity of thermal radiation along the line of sight, its influence on emissivity is minimal. Given the low overall evaporation rates in our experiments, any impact of vapor transmissivity on the temperature measurements is considered negligible.

#### Differential method

This method follows the same principle as the blackbody radiator approach but incorporates an additional reference measurement. To determine $\epsilon _{\text {eq}}$, two reference measurements are taken by heating the porous, liquid-filled medium to $T_{\text {ref},1}$ and $T_{\text {ref},2}$, while keeping all external flow conditions constant. This results in the following set of equations:

10 $$\begin{aligned} \sigma T_{\text {cam},1}^4&= \tau _{\text {eq}} (\epsilon _{\text {eq}} \sigma T_{\text {ref},1}^4) + I_{\text {noise}} \, , \end{aligned}$$

11 $$\begin{aligned} \sigma T_{\text {cam},2}^4&= \tau _{\text {eq}} (\epsilon _{\text {eq}} \sigma T_{\text {ref},2}^4) + I_{\text {noise}} \, . \end{aligned}$$

The above relation can now be rearranged by subtracting the two reference equations, allowing for a direct calculation of $\epsilon _{\text {eq}}$ from the two reference measurements as follows:

12 $$\begin{aligned} \epsilon _{\text {eq}} = \frac{T_{\text {cam},2}^4-T_{\text {cam},1}^4}{\tau _{\text {eq}}(T_{\text {ref},2}^4 - T_{\text {ref},1}^4)} \, . \end{aligned}$$

Also, as in the method of the blackbody radiator, the same assumptions are applied. It is assumed that both $\epsilon _{\text {eq}}$ and $I_{\text {noise}}$ remain nearly constant between the two reference cases, which is reasonable for small temperature differences. Furthermore, due to the minimal temperature change between the reference and measurement cases, it is assumed that the optical path transmission remains unchanged and that, as above, $\tau _{\text {eq}} = 1$. By using two reference cases with slightly varying temperatures and identical boundary conditions, it is possible to neglect all directional influences in the emissivity determination. Given the equivalent emissivity, the relationship from Eq. 9 can be used and the temperature distribution during a transpiration cooling experiment without heating can be evaluated.

13 $$\begin{aligned} T_{\text {exp}} = \root 4 \of {T_{\text {ref},1}^4 -\frac{T_{\text {cam},1}^4-T_{\text {cam, exp}}^4}{\tau _{\text {eq}} \epsilon _{\text {eq}}}} \end{aligned}$$

Looking at Eq. 12 and Eq. 13, it is obvious that the choice of $\tau _{\text {eq}}$ has no influence on the determined temperature. This parameter therefore only has an influence on the distribution of the equivalent emissivity $\epsilon _{\text {eq}}$.

## Data Availability

The datasets generated or analyzed during this current study are available from the corresponding author on reasonable request.
